# Custom-Designed Glassy Carbon Tips for Atomic Force Microscopy

**DOI:** 10.3390/mi8090285

**Published:** 2017-09-20

**Authors:** Anna Zakhurdaeva, Philipp-Immanuel Dietrich, Hendrik Hölscher, Christian Koos, Jan G. Korvink, Swati Sharma

**Affiliations:** 1 Institute of Microstructure Technology, Karlsruhe Institute of Technology, Hermann-von-Helmholtz-Platz 1, 76334 Eggenstein-Leopoldshafen, Germany; anna.zakhurdaeva@kit.edu (A.Z.); philipp-immanuel.dietrich@kit.edu (P.-I.D.); hendrik.hoelscher@kit.edu (H.H.); christian.koos@kit.edu (C.K.); jan.korvink@kit.edu (J.G.K.); 2 Institute of Photonics and Quantum Electronics, Karlsruhe Institute of Technology, Engesserstrasse 5, 76131 Karlsruhe, Germany

**Keywords:** AFM tip, Carbon-NEMS, glassy carbon, pyrolysis, two-photon polymerization

## Abstract

Glassy carbon is a graphenic form of elemental carbon obtained from pyrolysis of carbon-rich precursor polymers that can be patterned using various lithographic techniques. It is electrically and thermally conductive, mechanically strong, light, corrosion resistant and easy to functionalize. These properties render it very suitable for Carbon-microelectromechanical systems (Carbon-MEMS) and nanoelectromechanical systems (Carbon-NEMS) applications. Here we report on the fabrication and characterization of fully operational, microfabricated glassy carbon nano-tips for Atomic Force Microscopy (AFM). These tips are 3D-printed on to micro-machined silicon cantilevers by Two-Photon Polymerization (2PP) of acrylate-based photopolymers (commercially known as IP-series resists), followed by their carbonization employing controlled pyrolysis, which shrinks the patterned structure by ≥98% in volume. Tip performance and robustness during contact and dynamic AFM modes are validated by morphology and wear tests. The design and pyrolysis process optimization performed for this work indicate which parameters require special attention when IP-series polymers are used for the fabrication of Carbon-MEMS and NEMS. Microstructural characterization of the resulting material confirms that it features a frozen percolated network of graphene sheets accompanied by disordered carbon and voids, similar to typical glassy carbons. The presented facile fabrication method can be employed for obtaining a variety of 3D glassy carbon nanostructures starting from the stereolithographic designs provided by the user.

## 1. Introduction

Advanced modes of Atomic Force Microscopy (AFM) combine localized material property characterization alongside topography mapping. The functional and most crucial part of this microscopy technique is the tip, which is a simple nanoelectromechanical systems (NEMS) device whose geometry, aspect ratio (AR), surface properties and chemical composition strongly influence the quality of any measurement [1,2]. The available variety of commercial AFM tips enables researchers to select the most suitable probe for their desired application area. In order to provide such a range of tips featuring different geometries and functionalities, numerous micro and nano fabrication methods combined with various materials such as silicon [3], diamonds [4], metals [5], hydrogels [6] and other photopolymers [7] have been employed for AFM tip fabrication. Each type of tip offers its own benefits, but also comes with an associated cost. The majority of AFM microscopists prefer to purchase their tips rather than fabricating them on their own. Consequently, only those types of tips that are proven to be useful for many users are commercialized. Specialized tips often incorporate a reasonable cost due to the cumbersome and low-yield techniques involved in their fabrication [4,8,9], which restricts their use to already optimized experiments rather than exploratory research. Another concern regarding the use of highly specialized AFM tips is that they are designed mainly for one application. For example, a high-AR tip specifically designed for the imaging of deep trenches might be less useful for harsh imaging conditions, biomedical applications, or wear tests.

Here we report on using glassy carbon as AFM tip fabrication material. Due to its excellent mechanical [10,11], electrical [12], and surface properties [13], it is of high interest for AFM applications where these features are desirable. Glassy carbon is an *sp*${}^{2}$-rich form of carbon that contains interconnected graphene fragments responsible for its electrical conductivity [14,15,16]. It is also well-known for its thermal conductivity [17], it can be functionalized using various chemical pathways [13], and it is corrosion resistant due to an unreactive surface [12,17]. Since glassy carbon is obtained by pyrolysis (inert heating at 900 °C) of polymers, the precursor of the final structure can be obtained by several state-of-the-art fabrication techniques. The challenge at the nano-scale, however, is that only a handful of techniques allow for a controlled 3D fabrication, and those which do, may not use polymers that can be carbonized or retain their shape after pyrolysis. Notably, the polymer’s chemical composition strongly influences its carbon-conversion behaviour. For example, a large fraction of unsaturated bonds, a non-carbon backbone, inability to form a sufficient number of C–C bonds during heat-treatment, or an abundance of oxygen or halide atoms in a polymer, may lead to an unsuccessful pyrolysis resulting in a distorted or porous structure, or an extremely low carbon yield [14]. During pyrolysis, the morphology of a structure can only be preserved if the glass transition temperature $\left(T_{g}\right)$ of the carbonizing matrix increases faster than the rate of the chemical reactions responsible for polymer’s thermal transformation [18,19,20]. In other words, if at any given point during pyrolysis the material experiences a temperature above its glass transition temperature, it will go though a rubbery state and will lose its pre-patterned shape [21]. Importantly, the composition of the carbonizing material continuously changes during pyrolysis due to its thermochemical decomposition followed by C–C bond formation, which in turn causes its $T_{g}$ to change.

To address the aforementioned fabrication challenge we utilized Two-Photon Polymerization (2PP) process on a commercial 3D printer that operates with specialized inks, namely IP- series polymers, for the fabrication of cone-shaped structures prior to pyrolysis. Two such polymers, IP-Dip and IP-L were 3D-printed in the reported work. This technique is capable of patterning a range of user-defined complex 3D structures, however, there are only a few reported examples of carbonization of the polymers that are compatible with it [7,11]. To our knowledge, there is no established protocol to design shapes that can be successfully carbonized. Based on our observations, any free-standing structures (such as suspended bridges) fabricated in IP-resists may collapse during pyrolysis. As we show in this contribution, conical shapes typically retain their morphology despite significant structural shrinkage and display very good adhesion to silicon substrates owing to a wider supporting base. In order to determine the optimum shape, size and design we fabricated and characterized over 100 tips. Volume shrinkage upon carbonization, as well as the tip performance, were highly consistent in all cases.

Most carbonizable polymer precursors yield glassy carbon when pyrolyzed at 900 °C [14]. However, we performed microstructural characterization with the aid of Transmission Electron Microscopy (TEM) analysis and Raman spectroscopy specific to IP-Dip derived carbon since, to our knowledge, no such study has been previously reported. These tests will lay the foundation of further use of IP-series polymers in the field of Carbon-MEMS fabrication.

## 2. Materials and Methods

### 2.1. 2PP Fabrication

Various cone-shaped tips (heights between 10 and 100 µm and AR of 1, 2 or 3) were first patterned on silicon wafers in IP-Dip and IP-L photoresists (Nanoscribe GmbH) with a Nanoscribe 2PP set-up equipped with galvo scanning mirrors. After process optimization, only those geometries with a high printing yield, and no shape distortion during pyrolysis, were written on tipless silicon cantilevers (NanoAndMore GmbH, NSC37/38 series) without any additional surface treatment or adhesive layer. Fabrication was carried out in galvo mode with a laser power of 30 or 33.6 mW (homogeneous laser power distribution for all layers), a 25 mm/s writing speed, and a hatching and slicing distance of 200 nm. Tips were post-processed with UV/ DUV (200 mJ/cm${}^{2}$) followed by post-exposure bake (75 °C for 15 min) prior to their carbonization.

### 2.2. Pyrolysis

Pyrolysis was performed in a tube furnace (Heraeus GmbH, Hanau, Germany) under vacuum (10${}^{-6}$ atm.) environment at the maximum pyrolysis temperature of 900 °C [11]. Cantilevers were placed with tip-apex facing down in order to avoid bending.

### 2.3. AFM

All AFM measurements were conducted with a Dimension Icon AFM (Bruker) in a cleanroom environment. Surface morphology tests on AFM calibration gratings CS-20NG (Budget Sensors) and TGT1 (NT-MDT), multilayer carbon fiber mat and electroplated copper surface were performed in dynamic mode using glassy carbon tips with height of 5 µm and AR of 2. Tip wear tests were conducted on electroplated copper surface in contact mode at two scan velocities of 10 µm/s and 100 µm/s. Single scan was 2 µm by 2 µm and tip-apex profiles were obtained by blind reconstruction (SPIP software, Image Metrology A/S) using the data obtained from calibration grating TGT1 after each scan. During wear tests, images of the calibration grating TGT1 were obtained in dynamic mode in order to prevent the tested tips from any additional mechanical wear. Scanning Electron Microscopy (SEM) images of used tips were taken after a 6.1 mm scan distance for the two aforementioned scan velocities.

### 2.4. SEM

SEM investigations before and after pyrolysis were conducted on a ZEISS Supra 60VP SEM machine (Carl Zeiss AG, Oberkochen, Germany).

### 2.5. TEM

TEM investigations were performed on a FEI Titan 80-300 machine. For this purpose, a micropillar in the same dimension range as the tips used for the AFM wear tests was fabricated using 2PP, and was pyrolyzed using the same heat-treatment program. A slice of this carbonized, pillar obtained by Focused Ion Beam (FIB) milling, was imaged under the TEM.

### 2.6. Raman Spectroscopy

Raman spectroscopy of various pyrolyzed IP-Dip films (100 µm × 100 µm × 2 µm dimension.) was carried out on a Bruker Senterra confocal Raman-microscope using a 532 nm excitation wavelength. An average of 5 different spectra was taken for analysis.

## 3. Results and Discussion

### 3.1. Tip Design and Fabrication

**Figure 1:**
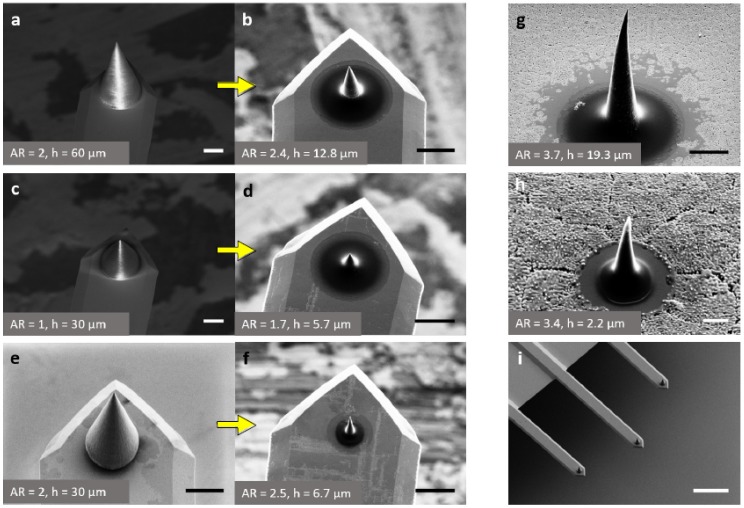
(**a**–**d**) SEM images of IP-Dip tips; and (**e**,**f**) IP-L tip before and after carbonization (indicated by the left-to-right arrows). Examples of (**g**) high aspect ratio; and (**h**) ultra-small carbon tips fabricated on silicon wafers; (**i**) Typical glassy carbon tips fabricated on a chip with three (previously) tip-less cantilevers. The aspect ratio and height *h* of each tip is given at the lower left of the SEM images. Scale bars: (**a**–**g**) 10 µm; (**h**) 1 µm; (**i**) 100 µm.

Typical SEM micrographs of various tips are shown in Figure 1. Figure 1a–d are micrographs of the *same* tips (fabricated using IP-Dip) before (left) and after (right) carbonization. The considerable size reduction caused by the pyrolysis process is obvious. The apparent volume shrinkage, as calculated from the SEM images assuming a conical shape, was approximately 98% (arithmetic mean of the observed shrinkage for all reported tips). Figure 1e,f represent a tip written in IP-L resist before and after carbonization. Using layer-by-layer fabrication, 9–10 tips per hour could be printed with approximately 90% yield. Radii of the tips printed on cantilevers were in the 200–250 nm before and 60–90 nm range after pyrolysis. While the entire conical structure shrinks by ≥98%, the size of the tip-apex only reduces by approximately 70%. This could be due to the fact that the carbonizing material goes through a semi-solid state during its pyrolysis, causing the tip-apex to slightly flatten.

Notably, tips without any post-processing tend to contain parts of uncrosslinked polymers that makes them mechanically weaker, vulnerable to chemical attacks and occasionally porous. Such tips typically feature a non-uniform shrinkage during pyrolysis and consequently yield a distorted structure. Tips with an initial AR of more than 3 often displayed a slight bending despite post-processing (see Figure 1g,h) resulting in a low fabrication yield. We, therefore, did not consider them for the subsequent AFM experiments reported here. However, they are potentially useful for the analysis of deep trenches [7]. We also fabricated ultra-small tips with a base diameter less than a micron and an AR greater than 3 to demonstrate the capabilities of the discussed fabrication approach. Such tips turned out to be extremely sharp but their yield was only 10–15%, since many of them practically disappeared due to the high volume shrinkage. One such miniature tip that was successfully carbonized is shown in Figure 1h. Figure 1i shows a chip with three cantilevers of different lengths (250 ± 5, 300 ± 5 and 350 ± 5 µm, respectively) used in our experiments. The change of their resonance frequencies before and after tip fabrication was negligible due to the extremely light weight of glassy carbon.

### 3.2. AFM Measurements

#### 3.2.1. Surface Morphology Tests

**Figure 2:**
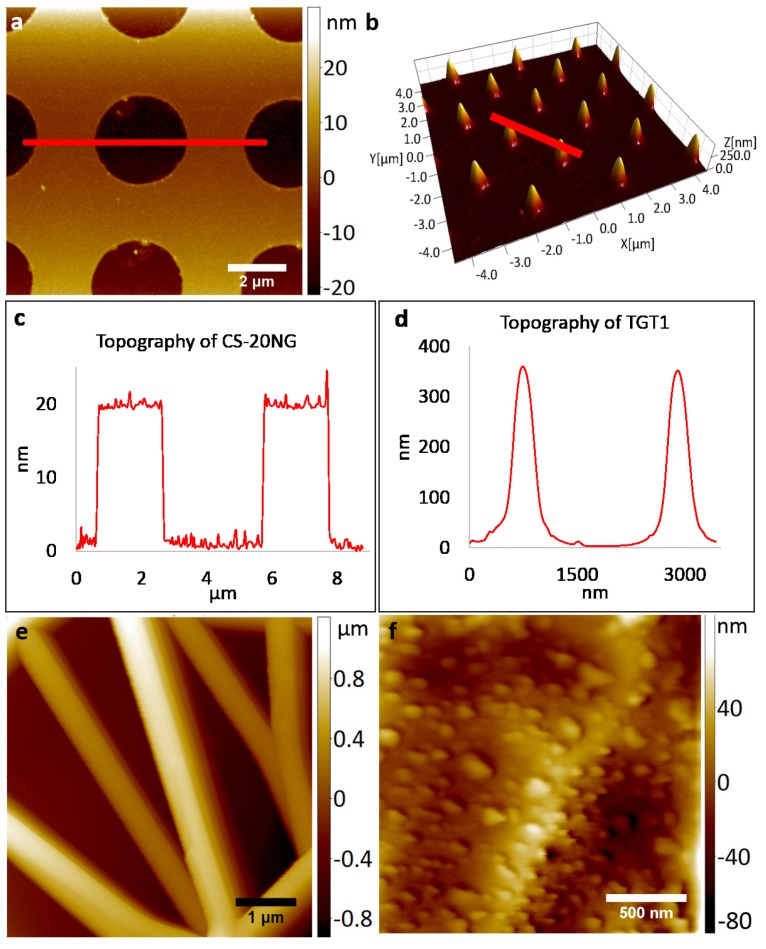
Atomic Force Microscopy (AFM) images recorded with glassy carbon tips. (**a**) Calibration grating CS-20NG; (**b**) calibration grating TGT1, with cross-sections indicated by solid lines in (**c**,**d**), respectively. AFM images of (**e**) a carbon nanofiber mat and (**f**) electroplated copper.

AFM images of various test surfaces obtained using glassy carbon tips are shown in Figure 2. Characteristic features of calibration gratings CS-20NG (circular patterns with 20 nm depth), and TGT1 (spikes of 300–500 nm height) can be clearly observed in Figure 2a,b. Corresponding cross-sectional profiles of these images (Figure 2c,d) demonstrate the details that can be efficiently resolved. The average measured height of a TGT1 spike (Figure 2b) was 350 nm, which corresponds to its vendor supplied values. We also successfully acquired images of samples with variable depth profiles such as a carbon fiber mat as shown in Figure 2e. The measured depth in this case was in line with the overall dimensions and sharpness of the tip used for analysis. Finally, Figure 2f) represents the AFM image of an electroplated copper surface where the achieved lateral resolution is approximately 40 nm. Notably, both vertical and lateral resolution can be further improved if a taller or sharper carbon tip is used for imaging. The purpose of the current study was to demonstrate that the tips obtained using reported fabrication technique can be successfully integrated with a standard AFM set-up and are fully functional.

#### 3.2.2. Wear Tests

**Figure 3:**
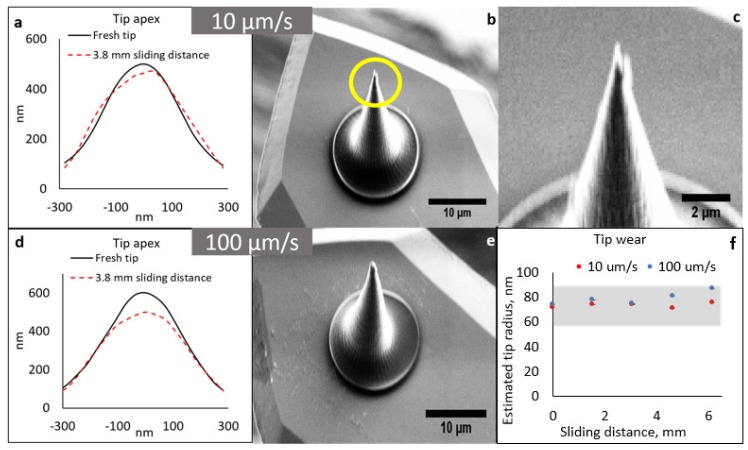
Wear tests for glassy carbon tips. (**a**) Pre- and post-wear tip-apex profiles and (**b**) corresponding post-wear SEM micrograph at 10 µm/s scan velocity. Magnified image of the apex of the tip in (**b**) (highlighted by a circle) is shown in (**c**); (**d**) pre- and post-wear tip-apex profiles and (**e**) corresponding post-wear SEM micrograph at 100 µm/s scan velocity; (**f**) Measured change in tip radii at 10 and 100 µm/s scan velocities at various sliding distances.

Tip-apex profiles and SEM micrographs after performing the wear tests at scan velocities of 10 µm/s and 100 µm/s, taken after a 6.1 mm scan distance are shown in Figure 3a–e. Figure 3c is a high magnification view of the post-wear tip shown in Figure 3b. Evidently, the tip-apex geometries remained unchanged and the tips did not exhibit any detachment from the cantilever surface after the tests. Occasionally, minor contamination from the test surface could be observed on the tip-apex (Figure 3c). Tip radii measured at various scan distance increments are shown in Figure 3f. As it can be observed, the tip-radii were comparable before and after the scans (in 60–90 nm range represented by the gray band in Figure 3f). Minor variations may result from radius measurement errors or possible contamination at the tip-apex. This test substantiates the fact that, due to its superior mechanical properties [10,11], glassy carbon is a very suitable tip material for contact mode AFM operations.

### 3.3. Material Characterization

**Figure 4:**
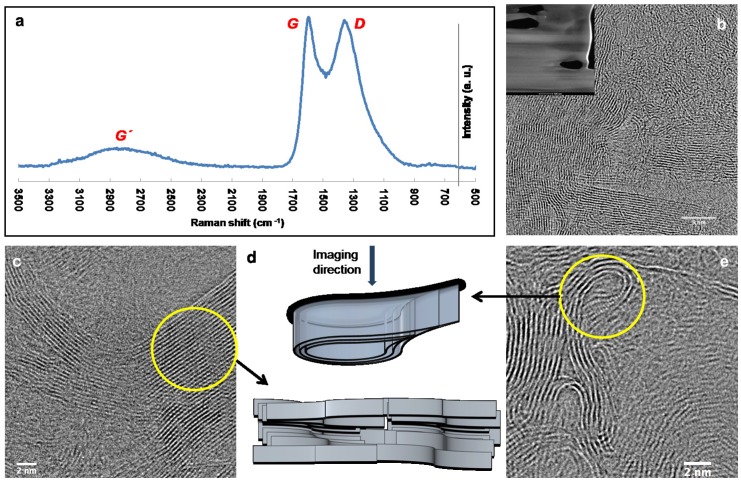
Microstructural characterization of IP-Dip-derived glassy carbon. (**a**) Raman spectrum; (**b**) Transmission Electron Microscopy (TEM) image showing the microstructure observed in the majority of the material (inset: Focused Ion Beam (FIB) milled sample used for TEM analysis); (**c**) TEM image featuring a graphitizing region; (**d**) Pictorial representation of possible 3D arrangement of graphene sheets in the material (locations highlighted by circles); (**e**) TEM image featuring strongly folded graphene sheets. Scale bars: (**b**) 5 nm; (**c**,**e**) 2 nm.

Raman spectrum and TEM images of IP-derived carbon with a plausible 3D arrangement of the graphene sheets in the material are shown in Figure 4.The Raman spectrum, presented in Figure 4a features clear *D* (disordered) and *G* (graphitic) Raman shift peaks characteristic of glassy carbons [16,22] with the highest intensities at 1361 and 1596 cm${}^{-1}$ respectively. The broad peak in the 2500–3100 cm${}^{-1}$ region is the 2D ($G^{\prime }$) band exhibited by most *sp*${}^{2}$-rich carbon materials during Raman investigations [23].

During TEM investigations the material featured a microstructure similar to that of commercial glassy carbons [14,16] for the most part, which can be observed in the image shown in Figure 4b. The FIB milled slice of the sample, which was analyzed by TEM, can be seen in the inset of Figure 4b. Some regions also resembled a ‘graphitizing’ type of pyrolytic carbon [24], exhibiting graphene sheets stacked with a preferred orientation (Figure 4c). Polymers such as anthracene yield this type of carbon on pyrolysis [15]. Another interesting observation was the identification of completely folded (U-turned) graphene sheets (Figure 4d). We speculate that this results either from the layer-by-layer fabrication (returning laser beam near the edges), or from the thermal stresses caused by FIB milling. Graphene sheets tend to fold and attain a more closely packed structure at higher temperatures to reduce their surface energy [15]. Such behaviour is frequently observed in carbons that have been exposed to harsh conditions such as arc evaporation [25]. Ion-beam induced stresses should, therefore, not be completely overlooked, which is often the case since FIB milling is a standard technique for TEM sample preparation [26]. In Figure 4d we propose possible 3D arrangements of graphene sheets in specific regions of IP-dip-derived carbon that cannot be observed directly in TEM images due to the lack of depth perception of this technique.

Here it is important to note that TEM and Raman spectroscopy could not be performed directly on AFM tips due to the specific sample preparation requirements of TEM and Raman spectroscopy. We therefore fabricated micropillars and films of the same size range as the tips employing identical fabrication and pyrolysis conditions in order to achieve comparable crystallinity in the material. TEM analysis entailed an additional step of FIB milling of the micropillar [27] in order to obtain a slice of the material thinner than 20 nm for imaging. Based on these microstructural analyses we conclude that the physicochemical properties of IP-Dip, pyrolyzed at 900 °C, are in the same range as graphitic glassy carbons.

IP-Dip is a very recent polymer in the field of Carbon-MEMS. Microstructural analysis of this material will be helpful for finding further applications. By modifying pyrolysis conditions it is possible tune the fraction of *sp*${}^{2}$ hybridized atoms in the final material [16,20], which in turn may improve its electrical and electrochemical properties suitable for a desired application. Glassy carbon is also defect-rich [28] that leads to its interesting surface properties. All described features make the material attractive for many engineering challenges. Common polymers utilized for Carbon-MEMS/NEMS fabrication are SU-8, polyacrylonitrile, or hydrogels that typically yield more than 30% carbon after pyrolysis [14]. IP-Dip, according to the vendor supplied datasheet, contains 60–80% of 2-(hydroxymethyl)-2-[[(1-oxoallyl)oxy]methyl]-1,3-propanediyl diacrylate (common name: Pentaerythritol triacrylate), which has a large fraction of bonded oxygen. It is therefore not surprising to see the huge volume loss during pyrolysis, likely due to the formation of CO${}_{2}$ and CO during the heat-treatment [14]. Our study demonstrated that IP-series polymers, despite of their relatively low carbon content, can still be used for carbon-based micro- and nano-fabrication. Indeed, one can take advantage of this feature to obtain much higher structural shrinkage and fabricate ultra-small NEMS-components, even when the fabrication technique used for initial polymer patterning is only capable of micro-scale fabrication. Notably, while the microstructure of the carbon obtained from IP-Dip is similar to that of SU-8 derived carbon, its pyrolysis behaviour ($T_{g}$ increment pattern during pyrolysis) is significantly different. Other polymers, such as some hydrogels that have already been explored for AFM tip fabrication [6], can also benefit from pyrolysis. Such polymer nano-tips are expected to feature atomic AFM resolution after their conversion into glassy carbon.

## 4. Conclusions

In summary, we presented a facile method based on a one-step conversion of micro to nano-scale features for the fabrication of robust, multipurpose tips that are ready to use in standard AFM applications. Our study shows that IP-series polymers are promising candidates in carbon device research with an already proven compatibility with 2PP and high structural shrinkage on pyrolysis. Although the current fabrication process relies on a serial, layer-by-layer printing methodology, it is reasonably fast with a high yield, allowing for batch fabrication of on-demand glassy carbon tips. We have also demonstrated the fabrication of ultra-sharp and tall glassy carbon tips employing the presented method, which are expected to result in a much higher lateral resolution. Some applications might benefit from the chemical inertness and hydrophobicity of the glassy carbon surface, which renders it less susceptible to contamination or corrosion. Their mechanical robustness makes the tips useful candidates for nano-scratching and AFM-assisted lithography. Other specialized AFM modes that might benefit from these tips are conductive-AFM and scanning thermal microscopy.
